# (3*S*,4*S*)-3-Ethyl-4-hydr­oxy-3-(3-methoxy­phen­yl)-1-methyl­azepan-1-ium d-tartrate dihydrate

**DOI:** 10.1107/S1600536808005898

**Published:** 2008-04-02

**Authors:** Xing-Hai Wang, Bo Chao, Zhui-Bai Qiu

**Affiliations:** a Department of Medicinal Chemistry, School of Pharmacy, Fudan University, 138 Yixueyuan Road, Shanghai 200032, People’s Republic of China

## Abstract

In the title compound, C_16_H_26_NO_2_^+^·C_4_H_5_O_6_^−^·2H_2_O, a meptaz­inol derivative, three C atoms of the azepane ring are disordered over two positions, with site-occupancy factors of 0.80 and 0.20; the major disorder component adopts a twist-chair conformation, while the minor component has a chair conformation. The benzene ring is axially substituted on the heterocyclic ring, resulting in a folded conformation of the cation. The absolute configuration was determined with reference to d-tartaric acid. The crystal structure is stabilized by an extensive network of intra- and inter­molecular O—H⋯O hydrogen bonds.

## Related literature

For the synthesis of the racemate of the title compound, see: Hao *et al.* (2005 ▶). For conformational studies of seven-membered rings, see: Eliel *et al.* (1994 ▶); Entrena *et al.* (2005 ▶). For the analgesic activity and clinical use of meptazinol, see: Holmes (1985 ▶). For related literature, see: Bill *et al.* (1983 ▶).
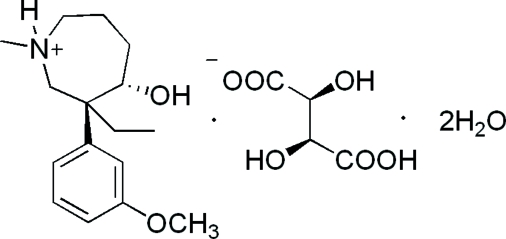


## Experimental

### 

#### Crystal data

C_16_H_26_NO_2_^+^·C_4_H_5_O_6_^−^·2H_2_O
*M* *_r_* = 449.49
Orthorhombic, 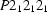

*a* = 7.146 (3) Å
*b* = 10.812 (4) Å
*c* = 29.338 (11) Å
*V* = 2266.7 (15) Å^3^
*Z* = 4
Mo *K*α radiation
μ = 0.10 mm^−1^
*T* = 293 (2) K
0.20 × 0.15 × 0.12 mm

#### Data collection

Bruker SMART APEX CCD area-detector diffractometer
Absorption correction: multi-scan (*SADABS*; Sheldrick, 1996 ▶) *T*_min_ = 0.979, *T*_max_ = 0.988
11334 measured reflections
2855 independent reflections
2173 reflections with *I* > 2σ(*I*)
*R*_int_ = 0.076

#### Refinement

*R*[*F* ^2^ > 2σ(*F* ^2^)] = 0.061
*wR*(*F* ^2^) = 0.163
*S* = 1.04
2855 reflections
319 parameters
19 restraints
H atoms treated by a mixture of independent and constrained refinement
Δρ_max_ = 0.43 e Å^−3^
Δρ_min_ = −0.26 e Å^−3^

### 

Data collection: *SMART* (Bruker, 2000 ▶); cell refinement: *SAINT* (Bruker, 2000 ▶); data reduction: *SAINT*; program(s) used to solve structure: *SHELXTL* (Sheldrick, 2008 ▶); program(s) used to refine structure: *SHELXTL*; molecular graphics: *SHELXTL* and *ORTEP-3* (Farrugia, 1997 ▶); software used to prepare material for publication: *SHELXTL* and local programs.

## Supplementary Material

Crystal structure: contains datablocks global, I. DOI: 10.1107/S1600536808005898/wn2243sup1.cif

Structure factors: contains datablocks I. DOI: 10.1107/S1600536808005898/wn2243Isup2.hkl

Additional supplementary materials:  crystallographic information; 3D view; checkCIF report

## Figures and Tables

**Table 1:** Hydrogen-bond geometry (Å, °)

*D*—H⋯*A*	*D*—H	H⋯*A*	*D*⋯*A*	*D*—H⋯*A*
O5—H5⋯O4	0.82	2.15	2.623 (5)	116
O6—H6⋯O9	0.82	1.91	2.641 (5)	149
O9—H9*X*⋯O2	0.83 (2)	2.00 (2)	2.823 (5)	171 (6)
O10—H10*X*⋯O5	0.82 (2)	1.98 (2)	2.790 (5)	173 (6)
C7*A*—H7*A*⋯O1	0.97	2.56	3.149 (8)	119
C6*B*—H6*B*1⋯O1	0.97	1.91	2.46 (3)	113
O9—H9*Y*⋯O7^i^	0.83 (2)	1.85 (2)	2.680 (5)	171 (7)
O3—H3⋯O8^i^	0.82	1.73	2.516 (4)	160
O5—H5⋯O10^ii^	0.82	2.27	2.983 (5)	145
O10—H10*Y*⋯O4^iii^	0.82 (2)	2.02 (4)	2.739 (5)	145 (6)
C16—H16⋯O7^iv^	0.93	2.50	3.417 (6)	171
C7*B*—H7*B*1⋯O6^iv^	0.97	2.51	3.176 (7)	126
C6*B*—H6*B*2⋯O1^v^	0.97	2.39	3.056 (17)	125

Symmetry codes: (i) 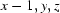; (ii) 
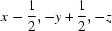
; (iii) 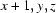; (iv) 
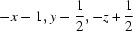
; (v) 
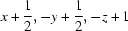
.

**Fig. 1.**
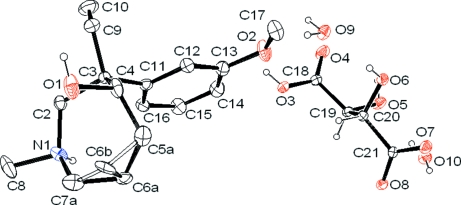
The molecular structure of the title compound, showing displacement ellipsoids at the 20% probability level. Hydrogen atoms are shown as spheres of arbitrary radius. Both azepane ring conformations are depicted; the minor chair conformation is drawn with open bonds. H atoms bonded to the C atoms of the azepane unit have been omitted for clarity.

**Fig. 2.**
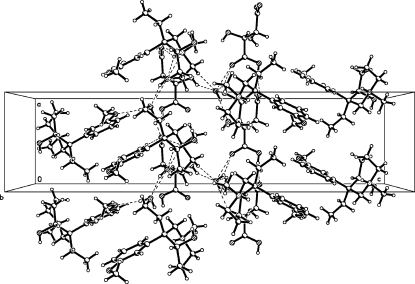
A view of the crystal packing, showing the hydrogen-bonding network (dashed lines). Only the twist-chair conformation of the azepane ring is shown.

## supplementary crystallographic information

### Comment

Meptazinol is a selective µ agonist with additional central anticholinergic activity, which has been used for treating pain associated with labour and kidney problems (Holmes, 1985). Many studies have shown meptazinol to have an advantage over other opioid analgesics because of its lack of adverse cardiorespiratory effects and low addiction liability (Bill *et al.*, 1983); this makes it an ideal precursor for further investigation. During the course of our structural optimization of meptazinol, the title compound was synthesized by introducing an OH group at the 4-position, followed by resolution with D-tartaric acid.

The absolute configuration of the azepane ring atoms is N1(*R*), C3(S) and C4(S), according to the reference molecule D-tartaric acid. The O—H functionalities, carboxyl group and H_2_O are known to be efficient donor and acceptor groups for hydrogen bonding, and they form an extensive hydrogen-bond network, which stabilizes the structure. The 3-methoxyphenyl substituent at C3 is *trans* to the OH group at C4 and *cis* to the N—H bond, resulting in a folded conformation of the cation.

The major disorder component adopts a twist-chair conformation, while the minor component has a chair conformation. The twist-chair conformation of seven-membered rings is known to be more stable than the chair conformation (Entrena *et al.*, 2005). Thus, the relative proportion of both conformers observed within the crystal structure may reflect the statistical partitioning of the two populations of azepane structures corresponding to different energetic states.

### Experimental

The title compound was prepared by standard procedures upon optical resolution of the racemate with D-tartaric acid. The synthesis of the racemic compound was described by Hao *et al.* (2005).

### Refinement

The H atoms bonded to N and O in the azepane ring, also the water hydrogen atoms were located in difference maps and refined with restraints: N—H = 0.89 (2) Å and O—H = 0.82 (2) and 0.83 (2) Å. The H atoms attached to O in the anion and all carbon-bound H atoms were placed in calculated positions and refined as riding; O—H = 0.82 and C—H = 0.93 - 0.98 Å; *U*_iso_(H) = *xU*_eq_(parent atom) where *x* = 1.5 for O and 1.2 for C. In the cation, three C atoms with attached H atoms are disordered over two positions; the site occupancy factors are 0.80 and 0.20.

### Crystal data

**Table:**

C_16_H_26_NO_2_^+^·C_4_H_5_O_6_^–^·2H_2_O	*F*_000_ = 968
*M**_r_* = 449.49	*D*_x_ = 1.317 Mg m^−^^3^
Orthorhombic, *P*2_1_2_1_2_1_	Mo *K*α radiation λ = 0.71073 Å
Hall symbol: P 2ac 2ab	Cell parameters from 1000 reflections
*a* = 7.146 (3) Å	θ = 2.8–22.5º
*b* = 10.812 (4) Å	µ = 0.11 mm^−^^1^
*c* = 29.338 (11) Å	*T* = 293 (2) K
*V* = 2266.7 (15) Å^3^	Prismatic, colorless
*Z* = 4	0.20 × 0.15 × 0.12 mm

### Data collection

**Table:**

Bruker SMART APEX CCD area-detector diffractometer	2855 independent reflections
Radiation source: fine-focus sealed tube	2173 reflections with *I* > 2σ(*I*)
Monochromator: graphite	*R*_int_ = 0.076
*T* = 293(2) K	θ_max_ = 27.1º
φ and ω scans	θ_min_ = 1.4º
Absorption correction: multi-scan(SADABS; Sheldrick, 1996)	*h* = −9→4
*T*_min_ = 0.979, *T*_max_ = 0.988	*k* = −13→13
11334 measured reflections	*l* = −37→37

### Refinement

**Table:**

Refinement on *F*^2^	Secondary atom site location: difference Fourier map
Least-squares matrix: full	Hydrogen site location: inferred from neighbouring sites
*R*[*F*^2^ > 2σ(*F*^2^)] = 0.061	H atoms treated by a mixture of independent and constrained refinement
*wR*(*F*^2^) = 0.163	*w* = 1/[σ^2^(*F*_o_^2^) + (0.0771*P*)^2^] where *P* = (*F*_o_^2^ + 2*F*_c_^2^)/3
*S* = 1.04	(Δ/σ)_max_ < 0.001
2855 reflections	Δρ_max_ = 0.43 e Å^−^^3^
319 parameters	Δρ_min_ = −0.26 e Å^−^^3^
19 restraints	Extinction correction: none
Primary atom site location: structure-invariant direct methods	

### Special details

**Table:**

Geometry. All e.s.d.'s (except the e.s.d. in the dihedral angle between two l.s. planes) are estimated using the full covariance matrix. The cell e.s.d.'s are taken into account individually in the estimation of e.s.d.'s in distances, angles and torsion angles; correlations between e.s.d.'s in cell parameters are only used when they are defined by crystal symmetry. An approximate (isotropic) treatment of cell e.s.d.'s is used for estimating e.s.d.'s involving l.s. planes.
Refinement. Refinement of *F*^2^ against ALL reflections. The weighted *R*-factor *wR* and goodness of fit *S* are based on *F*^2^, conventional *R*-factors *R* are based on *F*, with *F* set to zero for negative *F*^2^. The threshold expression of *F*^2^ > σ(*F*^2^) is used only for calculating *R*-factors(gt) *etc*. and is not relevant to the choice of reflections for refinement. *R*-factors based on *F*^2^ are statistically about twice as large as those based on *F*, and *R*- factors based on ALL data will be even larger.

### Fractional atomic coordinates and isotropic or equivalent isotropic displacement parameters (Å^2^)

**Table:**

	*x*	*y*	*z*	*U*_iso_*/*U*_eq_	Occ. (<1)
O1	−1.0175 (7)	0.3641 (4)	0.46124 (12)	0.0684 (12)	
H1X	−1.114 (7)	0.405 (6)	0.457 (2)	0.14 (4)*	
O2	−0.7001 (6)	0.4436 (3)	0.25255 (10)	0.0628 (11)	
O3	−0.6276 (4)	0.1543 (3)	0.13391 (10)	0.0425 (7)	
H3	−0.7400	0.1418	0.1307	0.064*	
O4	−0.6399 (5)	0.2140 (4)	0.06130 (12)	0.0626 (10)	
O5	−0.2757 (4)	0.2069 (3)	0.05050 (10)	0.0487 (8)	
H5	−0.3605	0.2365	0.0352	0.073*	
O6	−0.3086 (4)	0.3996 (3)	0.11490 (10)	0.0430 (7)	
H6	−0.3766	0.4310	0.1342	0.065*	
O7	0.0443 (4)	0.3653 (3)	0.13158 (11)	0.0480 (8)	
O8	0.0205 (4)	0.1619 (3)	0.13574 (10)	0.0391 (7)	
O9	−0.6223 (5)	0.4527 (3)	0.15827 (12)	0.0473 (8)	
H9X	−0.651 (9)	0.457 (5)	0.1855 (9)	0.066 (17)*	
H9Y	−0.721 (6)	0.424 (7)	0.147 (2)	0.11 (3)*	
O10	0.0604 (5)	0.1148 (3)	0.01603 (11)	0.0472 (8)	
H10X	−0.033 (5)	0.142 (5)	0.0284 (18)	0.067 (18)*	
H10Y	0.121 (8)	0.135 (6)	0.0387 (14)	0.08 (2)*	
N1	−0.9584 (7)	0.0649 (3)	0.42832 (11)	0.0451 (10)	
H1	−0.925 (6)	0.018 (3)	0.4048 (10)	0.029 (11)*	
C2	−1.0991 (6)	0.1554 (4)	0.41055 (14)	0.0381 (9)	
H2A	−1.1746	0.1816	0.4363	0.046*	
H2B	−1.1814	0.1103	0.3902	0.046*	
C3	−1.0364 (6)	0.2740 (4)	0.38507 (13)	0.0340 (9)	
C4	−0.9242 (8)	0.3610 (4)	0.41797 (14)	0.0480 (12)	
H4	−0.9289	0.4447	0.4052	0.058*	
C5A	−0.7183 (8)	0.3276 (5)	0.42468 (16)	0.0575 (13)	0.80
H5A	−0.6738	0.3677	0.4522	0.069*	0.80
H5B	−0.6473	0.3609	0.3993	0.069*	0.80
C6A	−0.6754 (8)	0.1822 (6)	0.42849 (18)	0.0489 (15)	0.80
H6A	−0.6734	0.1480	0.3979	0.059*	0.80
H6B	−0.5509	0.1721	0.4412	0.059*	0.80
C7A	−0.8041 (9)	0.1098 (5)	0.4556 (2)	0.0691 (15)	0.80
H7A	−0.8522	0.1602	0.4803	0.083*	0.80
H7B	−0.7382	0.0401	0.4689	0.083*	0.80
C5B	−0.7183 (8)	0.3276 (5)	0.42468 (16)	0.0575 (13)	0.20
H5B1	−0.6630	0.2960	0.3968	0.069*	0.20
H5B2	−0.6463	0.3980	0.4353	0.069*	0.20
C6B	−0.730 (4)	0.2392 (14)	0.4568 (5)	0.049 (6)	0.20
H6B1	−0.7994	0.2782	0.4814	0.058*	0.20
H6B2	−0.6033	0.2304	0.4680	0.058*	0.20
C7B	−0.8041 (9)	0.1098 (5)	0.4556 (2)	0.0691 (15)	0.20
H7B1	−0.6980	0.0580	0.4477	0.083*	0.20
H7B2	−0.8358	0.0895	0.4869	0.083*	0.20
C8	−1.0659 (12)	−0.0250 (5)	0.45936 (18)	0.082 (2)	
H8A	−0.9931	−0.0986	0.4638	0.123*	
H8B	−1.1831	−0.0462	0.4454	0.123*	
H8C	−1.0888	0.0136	0.4883	0.123*	
C9	−1.2199 (7)	0.3436 (5)	0.37375 (18)	0.0567 (13)	
H9A	−1.2956	0.3471	0.4012	0.068*	
H9B	−1.1882	0.4280	0.3656	0.068*	
C10	−1.3354 (9)	0.2906 (7)	0.3367 (2)	0.0770 (18)	
H10A	−1.2604	0.2814	0.3097	0.116*	
H10B	−1.4386	0.3448	0.3303	0.116*	
H10C	−1.3821	0.2112	0.3459	0.116*	
C11	−0.9309 (6)	0.2535 (4)	0.34049 (13)	0.0338 (9)	
C12	−0.8603 (6)	0.3570 (4)	0.31824 (13)	0.0388 (10)	
H12	−0.8704	0.4346	0.3317	0.047*	
C13	−0.7751 (7)	0.3450 (5)	0.27617 (14)	0.0442 (10)	
C14	−0.7607 (8)	0.2313 (5)	0.25529 (15)	0.0528 (13)	
H14	−0.7037	0.2241	0.2269	0.063*	
C15	−0.8316 (8)	0.1289 (5)	0.27677 (14)	0.0523 (13)	
H15	−0.8234	0.0519	0.2628	0.063*	
C16	−0.9167 (6)	0.1400 (4)	0.31989 (13)	0.0412 (10)	
H16	−0.9634	0.0701	0.3344	0.049*	
C17	−0.6939 (10)	0.5617 (5)	0.27368 (17)	0.0643 (15)	
H17A	−0.8170	0.5840	0.2839	0.096*	
H17B	−0.6501	0.6220	0.2521	0.096*	
H17C	−0.6104	0.5590	0.2993	0.096*	
C18	−0.5553 (6)	0.1854 (4)	0.09532 (14)	0.0357 (9)	
C19	−0.3405 (5)	0.1845 (4)	0.09492 (14)	0.0348 (9)	
H19	−0.2979	0.1022	0.1042	0.042*	
C20	−0.2607 (5)	0.2792 (4)	0.12837 (13)	0.0311 (8)	
H20	−0.3144	0.2636	0.1586	0.037*	
C21	−0.0461 (6)	0.2685 (3)	0.13173 (12)	0.0304 (8)	

### Atomic displacement parameters (Å^2^)

**Table:**

	*U*^11^	*U*^22^	*U*^33^	*U*^12^	*U*^13^	*U*^23^
O1	0.102 (3)	0.054 (2)	0.0493 (19)	−0.015 (2)	0.036 (2)	−0.0117 (16)
O2	0.093 (3)	0.062 (2)	0.0337 (16)	−0.020 (2)	0.0192 (19)	−0.0047 (14)
O3	0.0267 (13)	0.0508 (18)	0.0499 (17)	−0.0006 (14)	−0.0032 (13)	0.0072 (15)
O4	0.0358 (17)	0.102 (3)	0.0504 (19)	−0.001 (2)	−0.0073 (16)	0.0149 (19)
O5	0.0355 (16)	0.071 (2)	0.0396 (16)	0.0106 (16)	0.0018 (14)	−0.0067 (15)
O6	0.0343 (16)	0.0337 (15)	0.0612 (19)	0.0056 (13)	0.0115 (15)	0.0052 (13)
O7	0.0328 (15)	0.0357 (16)	0.076 (2)	−0.0038 (14)	−0.0019 (17)	0.0016 (15)
O8	0.0268 (14)	0.0354 (15)	0.0550 (17)	0.0019 (12)	−0.0009 (13)	0.0041 (13)
O9	0.0393 (18)	0.059 (2)	0.0433 (19)	−0.0009 (16)	0.0073 (16)	−0.0042 (16)
O10	0.0456 (19)	0.0567 (19)	0.0392 (17)	0.0046 (18)	0.0032 (17)	−0.0048 (15)
N1	0.070 (3)	0.0334 (18)	0.0320 (18)	0.006 (2)	−0.007 (2)	−0.0040 (14)
C2	0.042 (2)	0.032 (2)	0.040 (2)	−0.0049 (19)	0.0031 (19)	0.0004 (17)
C3	0.034 (2)	0.0302 (19)	0.038 (2)	0.0035 (18)	0.0068 (19)	0.0033 (16)
C4	0.077 (3)	0.033 (2)	0.034 (2)	−0.005 (2)	0.024 (2)	−0.0071 (17)
C5A	0.055 (3)	0.078 (4)	0.039 (2)	−0.020 (3)	−0.007 (2)	−0.008 (2)
C6A	0.039 (3)	0.079 (4)	0.029 (3)	0.018 (3)	−0.008 (2)	0.004 (3)
C7A	0.074 (4)	0.061 (3)	0.073 (3)	0.007 (3)	−0.020 (3)	−0.002 (3)
C5B	0.055 (3)	0.078 (4)	0.039 (2)	−0.020 (3)	−0.007 (2)	−0.008 (2)
C6B	0.086 (18)	0.046 (9)	0.014 (8)	0.022 (10)	−0.020 (11)	−0.001 (8)
C7B	0.074 (4)	0.061 (3)	0.073 (3)	0.007 (3)	−0.020 (3)	−0.002 (3)
C8	0.147 (7)	0.049 (3)	0.050 (3)	0.009 (4)	0.039 (4)	0.014 (2)
C9	0.048 (3)	0.061 (3)	0.062 (3)	0.016 (3)	0.009 (3)	0.011 (2)
C10	0.050 (3)	0.104 (5)	0.077 (4)	0.020 (4)	−0.010 (3)	0.003 (4)
C11	0.030 (2)	0.042 (2)	0.0300 (18)	0.0037 (18)	−0.0032 (17)	0.0003 (16)
C12	0.046 (2)	0.043 (2)	0.0281 (19)	−0.003 (2)	−0.0044 (18)	−0.0038 (17)
C13	0.043 (2)	0.056 (3)	0.033 (2)	−0.005 (2)	−0.0041 (19)	0.0015 (19)
C14	0.059 (3)	0.068 (3)	0.032 (2)	0.000 (3)	0.006 (2)	−0.009 (2)
C15	0.068 (3)	0.051 (3)	0.038 (2)	0.000 (3)	0.002 (2)	−0.011 (2)
C16	0.048 (3)	0.039 (2)	0.036 (2)	0.006 (2)	0.0024 (19)	0.0006 (17)
C17	0.083 (4)	0.060 (3)	0.050 (3)	−0.017 (3)	0.003 (3)	0.004 (2)
C18	0.0300 (19)	0.037 (2)	0.040 (2)	0.0026 (18)	−0.004 (2)	−0.0023 (17)
C19	0.028 (2)	0.036 (2)	0.040 (2)	0.0058 (17)	−0.0012 (18)	0.0007 (17)
C20	0.0279 (19)	0.0302 (19)	0.0351 (19)	0.0021 (16)	0.0045 (17)	0.0018 (16)
C21	0.0273 (18)	0.033 (2)	0.0313 (18)	−0.0011 (17)	0.0036 (17)	0.0027 (15)

### Geometric parameters (Å, °)

**Table:**

O1—C4	1.434 (5)	C6A—H6A	0.9700
O1—H1X	0.83 (2)	C6A—H6B	0.9700
O2—C13	1.380 (6)	C7A—H7A	0.9700
O2—C17	1.420 (6)	C7A—H7B	0.9700
O3—C18	1.289 (5)	C6B—H6B1	0.9700
O3—H3	0.8200	C6B—H6B2	0.9700
O4—C18	1.207 (5)	C8—H8A	0.9599
O5—C19	1.404 (5)	C8—H8B	0.9599
O5—H5	0.8200	C8—H8C	0.9599
O6—C20	1.403 (5)	C9—C10	1.482 (8)
O6—H6	0.8200	C9—H9A	0.9700
O7—C21	1.230 (5)	C9—H9B	0.9700
O8—C21	1.252 (5)	C10—H10A	0.9599
O9—H9X	0.83 (2)	C10—H10B	0.9599
O9—H9Y	0.83 (2)	C10—H10C	0.9599
O10—H10X	0.82 (2)	C11—C16	1.371 (6)
O10—H10Y	0.82 (2)	C11—C12	1.391 (6)
N1—C7A	1.447 (7)	C12—C13	1.383 (6)
N1—C2	1.496 (6)	C12—H12	0.9300
N1—C8	1.538 (7)	C13—C14	1.377 (7)
N1—H1	0.890 (19)	C14—C15	1.371 (7)
C2—C3	1.551 (5)	C14—H14	0.9300
C2—H2A	0.9700	C15—C16	1.409 (6)
C2—H2B	0.9700	C15—H15	0.9300
C3—C11	1.526 (5)	C16—H16	0.9300
C3—C9	1.548 (7)	C17—H17A	0.9599
C3—C4	1.568 (6)	C17—H17B	0.9599
C4—C5A	1.528 (8)	C17—H17C	0.9599
C4—H4	0.9800	C18—C19	1.536 (6)
C5A—C6A	1.606 (8)	C19—C20	1.528 (6)
C5A—H5A	0.9700	C19—H19	0.9800
C5A—H5B	0.9700	C20—C21	1.541 (6)
C6A—C7A	1.446 (8)	C20—H20	0.9800
			
C4—O1—H1X	106 (6)	H8B—C8—H8C	109.5
C13—O2—C17	119.2 (4)	C10—C9—C3	116.2 (5)
C18—O3—H3	109.5	C10—C9—H9A	108.2
C19—O5—H5	109.5	C3—C9—H9A	108.2
C20—O6—H6	109.5	C10—C9—H9B	108.2
H9X—O9—H9Y	101 (6)	C3—C9—H9B	108.2
H10X—O10—H10Y	89 (5)	H9A—C9—H9B	107.4
C7A—N1—C2	119.1 (4)	C9—C10—H10A	109.5
C7A—N1—C8	105.4 (4)	C9—C10—H10B	109.5
C2—N1—C8	106.5 (4)	H10A—C10—H10B	109.5
C7A—N1—H1	115 (3)	C9—C10—H10C	109.5
C2—N1—H1	107 (3)	H10A—C10—H10C	109.5
C8—N1—H1	103 (3)	H10B—C10—H10C	109.5
N1—C2—C3	121.0 (4)	C16—C11—C12	119.1 (4)
N1—C2—H2A	107.1	C16—C11—C3	123.0 (4)
C3—C2—H2A	107.1	C12—C11—C3	117.7 (3)
N1—C2—H2B	107.1	C13—C12—C11	120.2 (4)
C3—C2—H2B	107.1	C13—C12—H12	119.9
H2A—C2—H2B	106.8	C11—C12—H12	119.9
C11—C3—C9	107.8 (3)	C14—C13—O2	115.9 (4)
C11—C3—C2	115.8 (3)	C14—C13—C12	121.0 (4)
C9—C3—C2	105.1 (4)	O2—C13—C12	123.1 (4)
C11—C3—C4	111.2 (3)	C15—C14—C13	119.3 (4)
C9—C3—C4	105.9 (4)	C15—C14—H14	120.4
C2—C3—C4	110.3 (3)	C13—C14—H14	120.4
O1—C4—C5A	109.8 (4)	C14—C15—C16	120.2 (4)
O1—C4—C3	108.7 (4)	C14—C15—H15	119.9
C5A—C4—C3	115.5 (4)	C16—C15—H15	119.9
O1—C4—H4	107.5	C11—C16—C15	120.3 (4)
C5A—C4—H4	107.5	C11—C16—H16	119.9
C3—C4—H4	107.5	C15—C16—H16	119.9
C4—C5A—C6A	115.1 (4)	O2—C17—H17A	109.5
C4—C5A—H5A	108.5	O2—C17—H17B	109.5
C6A—C5A—H5A	108.5	H17A—C17—H17B	109.5
C4—C5A—H5B	108.5	O2—C17—H17C	109.5
C6A—C5A—H5B	108.5	H17A—C17—H17C	109.5
H5A—C5A—H5B	107.5	H17B—C17—H17C	109.5
C7A—C6A—C5A	116.5 (5)	O4—C18—O3	126.3 (4)
C7A—C6A—H6A	108.2	O4—C18—C19	119.7 (4)
C5A—C6A—H6A	108.2	O3—C18—C19	114.0 (4)
C7A—C6A—H6B	108.2	O5—C19—C20	111.0 (3)
C5A—C6A—H6B	108.2	O5—C19—C18	109.6 (3)
H6A—C6A—H6B	107.3	C20—C19—C18	111.4 (3)
C6A—C7A—N1	111.2 (5)	O5—C19—H19	108.3
C6A—C7A—H7A	109.4	C20—C19—H19	108.3
N1—C7A—H7A	109.4	C18—C19—H19	108.3
C6A—C7A—H7B	109.4	O6—C20—C19	110.5 (3)
N1—C7A—H7B	109.4	O6—C20—C21	109.3 (3)
H7A—C7A—H7B	108.0	C19—C20—C21	111.2 (3)
H6B1—C6B—H6B2	105.5	O6—C20—H20	108.6
N1—C8—H8A	109.5	C19—C20—H20	108.6
N1—C8—H8B	109.5	C21—C20—H20	108.6
H8A—C8—H8B	109.5	O7—C21—O8	125.7 (4)
N1—C8—H8C	109.5	O7—C21—C20	117.3 (4)
H8A—C8—H8C	109.5	O8—C21—C20	117.0 (3)
			
C7A—N1—C2—C3	50.1 (6)	C4—C3—C11—C12	−48.4 (5)
C8—N1—C2—C3	168.8 (4)	C16—C11—C12—C13	−0.8 (6)
N1—C2—C3—C11	62.8 (5)	C3—C11—C12—C13	−175.4 (4)
N1—C2—C3—C9	−178.4 (4)	C17—O2—C13—C14	−174.2 (5)
N1—C2—C3—C4	−64.6 (5)	C17—O2—C13—C12	5.9 (8)
C11—C3—C4—O1	−172.6 (4)	C11—C12—C13—C14	0.9 (7)
C9—C3—C4—O1	70.6 (5)	C11—C12—C13—O2	−179.2 (4)
C2—C3—C4—O1	−42.7 (5)	O2—C13—C14—C15	179.8 (5)
C11—C3—C4—C5A	−48.7 (5)	C12—C13—C14—C15	−0.3 (7)
C9—C3—C4—C5A	−165.5 (4)	C13—C14—C15—C16	−0.5 (8)
C2—C3—C4—C5A	81.2 (4)	C12—C11—C16—C15	0.0 (7)
O1—C4—C5A—C6A	83.7 (5)	C3—C11—C16—C15	174.4 (4)
C3—C4—C5A—C6A	−39.7 (5)	C14—C15—C16—C11	0.6 (7)
C4—C5A—C6A—C7A	−41.9 (6)	O4—C18—C19—O5	−6.3 (6)
C5A—C6A—C7A—N1	88.8 (6)	O3—C18—C19—O5	174.0 (3)
C2—N1—C7A—C6A	−68.9 (6)	O4—C18—C19—C20	116.9 (5)
C8—N1—C7A—C6A	171.8 (5)	O3—C18—C19—C20	−62.9 (5)
C11—C3—C9—C10	50.3 (6)	O5—C19—C20—O6	57.8 (4)
C2—C3—C9—C10	−73.7 (5)	C18—C19—C20—O6	−64.6 (4)
C4—C3—C9—C10	169.4 (5)	O5—C19—C20—C21	−63.7 (4)
C9—C3—C11—C16	−107.3 (5)	C18—C19—C20—C21	173.9 (3)
C2—C3—C11—C16	10.1 (6)	O6—C20—C21—O7	13.9 (5)
C4—C3—C11—C16	137.1 (4)	C19—C20—C21—O7	136.2 (4)
C9—C3—C11—C12	67.2 (5)	O6—C20—C21—O8	−168.1 (3)
C2—C3—C11—C12	−175.4 (4)	C19—C20—C21—O8	−45.9 (5)

### Hydrogen-bond geometry (Å, °)

**Table:**

*D*—H···*A*	*D*—H	H···*A*	*D*···*A*	*D*—H···*A*
O5—H5···O4	0.82	2.15	2.623 (5)	116
O6—H6···O9	0.82	1.91	2.641 (5)	149
O9—H9X···O2	0.83 (2)	2.00 (2)	2.823 (5)	171 (6)
O10—H10X···O5	0.82 (2)	1.98 (2)	2.790 (5)	173 (6)
C7A—H7A···O1	0.97	2.56	3.149 (8)	119
C6B—H6B1···O1	0.97	1.91	2.46 (3)	113
O9—H9Y···O7^i^	0.83 (2)	1.85 (2)	2.680 (5)	171 (7)
O3—H3···O8^i^	0.82	1.73	2.516 (4)	160
O5—H5···O10^ii^	0.82	2.27	2.983 (5)	145
O10—H10Y···O4^iii^	0.82 (2)	2.02 (4)	2.739 (5)	145 (6)
C16—H16···O7^iv^	0.93	2.50	3.417 (6)	171
C7B—H7B1···O6^iv^	0.97	2.51	3.176 (7)	126
C6B—H6B2···O1^v^	0.97	2.39	3.056 (17)	125

Symmetry codes: (i) *x*−1, *y*, *z*; (ii) *x*−1/2, −*y*+1/2, −*z*; (iii) *x*+1, *y*, *z*; (iv) −*x*−1, *y*−1/2, −*z*+1/2; (v) *x*+1/2, −*y*+1/2, −*z*+1.
